# Stonebrood Disease—Histomorphological Changes in Honey Bee Larvae (*Apis mellifera*) Experimentally Infected with *Aspergillus flavus*

**DOI:** 10.3390/vetsci12020124

**Published:** 2025-02-04

**Authors:** Tammo von Knoblauch, Annette B. Jensen, Christoph K. W. Mülling, Anton Heusinger, Heike Aupperle-Lellbach, Elke Genersch

**Affiliations:** 1 LABOKLIN GmbH & Co.KG, Labor für Klinische Diagnostik, Steubenstraße 4, 97688 Bad Kissingen, Germany; knoblauch@laboklin.com (T.v.K.); heusinger@laboklin.com (A.H.); 2 Department of Plant and Environmental Sciences Section for Organismal Biology, University of Copenhagen, Thorvaldsensvej 40, 1871 Frederiksberg C, Denmark; abj@plen.ku.dk; 3 Institute of Veterinary Anatomy, Histology and Embryology, Faculty of Veterinary Medicine, Leipzig University, An den Tierkliniken 43, 04103 Leipzig, Germany; c.muelling@vetmed.uni-leipzig.de; 4 Department of Molecular Microbiology and Bee Diseases, Institute for Bee Research, Friedrich-Engels-Str. 32, 16540 Hohen Neuendorf, Germany; 5 Institute of Microbiology and Epizootics, Faculty of Veterinary Medicine, Freie Universität Berlin, Robert-von-Ostertag-Str. 7, 14163 Berlin, Germany

**Keywords:** insects, histopathology, intestinal infection, mycology, zoonotic infection, artificial rearing, fungal

## Abstract

Stonebrood (*Aspergillus* sp.) is a rare, poorly described disease of the Western honey bee (*Apis mellifera*) that can affect adult bees and brood. This study describes the pathogenesis using artificially reared pathogen-free *Apis mellifera* larvae, experimentally infected (5 × 10^2^ spores/larva) with *Aspergillus flavus*. Between days 1 and 5 p.i. (larval age 4 until 8 days), five uninfected control larvae, up to five infected living larvae, and up to five infected dead larvae were examined macroscopically. Subsequently, the larvae were photographed, fixed (4% formaldehyde), and processed for histological examination (hematoxylin–eosin stain, Grocott silvering). Sections were digitized, measured (area, thickness), and statistically analyzed. In total, 19 of the 43 collected infected larvae showed signs of infection (germinating spores/fungal mycelium): dead larvae (from day 2 p.i.) showed clear histological and macroscopic signs of infection, while larvae collected alive (from day 1 p.i.) were only locally affected. Infected larvae were significantly smaller (day 2 p.i.: *p* < 0.001, 4 p.i.: *p* < 0.01, 5 p.i.: *p* < 0.01) than uninfected larvae (control group). Our study shows that the pathogenesis of stonebrood is characterized by a short period between *Aspergillus* germination and the onset of disease (about one day), and a rapid larval death.

## Simple Summary

Stonebrood (*Aspergillus* sp.), a rare disease of the Western honey bee *Apis mellifera* affecting adult bees and brood, has been described only occasionally. This study analyzes the course of the disease (pathogenesis) using artificially reared pathogen-free *Apis mellifera* larvae experimentally infected with *Aspergillus flavus* and examined macroscopically and histologically. In 19 of the 43 larvae taken from the infected group, signs of infection in the form of germinating spores or fungal mycelium were detected. These larvae were significantly smaller than the larvae from the control group. Our study shows that the pathogenesis of stonebrood is characterized by a short period between *Aspergillus* germination and the onset of disease (about one day), and a rapid larval death.

## 1. Introduction

Stonebrood is a very rare fungal disease of the Western honey bee *Apis mellifera*, caused by various pathogen species from the genus *Aspergillus* [1,2,3]. Reports of clinically affected colonies are rare and sometimes very old [1,4]. As the name stonebrood suggests, the disease is regarded and, therefore, often exclusively described as a brood disease, although it also occurs in adult bees [1,2,3,5]. However, since affected adult bees probably die outside the colony, their disease status is usually not recognized as such [1,2,3].

As a quite rare honey bee disease, stonebrood never attracted much attention, despite the fact that it is the only zoonotic disease of honey bees [6,7,8]. Hence, in the rare case of a stonebrood-diseased colony, the beekeeper is at risk of inhaling spores which might result in severe health problems [9,10]. There are only very few scientific studies on stonebrood—much of the information on the pathogenesis of this disease is based on a larger study by Burnside from 1930, in which adult honey bees and larvae were experimentally infected (not under standardized conditions) [1]. Hence, the pathogenesis and etiology of stonebrood are still not fully understood [11,12].

It is assumed that stonebrood typically breaks out in weak colonies, and an outbreak is related to humid conditions in spring or occurs after warm summer rains [1]. However, in most cases, the infection does not lead to the death of a colony [1,13]. The pathogens have been detected in clinically healthy hives, their surroundings, and the gastrointestinal tract (alimentary tract) of adult bees and bee larvae [1,3,14,15]. Oral infection by conidia [1,5] in pollen [16,17,18,19] is discussed as the most likely source of entry. In adult bees, entry via the hemolymph, for example, in wounds and sporadic cases via the cuticle, has also been demonstrated experimentally [1].

Hard, mummified larvae, which give the disease its name, are characteristics of stonebrood [2,4,5,12]. While the stonebrood mummies initially look white and fluffy, the color changes depending on the *Aspergillus* species involved [1,5,12]. In the literature, it is described that infected and dead bee larvae were only partly removed by nurse bees, probably because, in contrast to chalkbrood, the fungal mycelium of the larvae infiltrates the cell wall, making mechanical removal more difficult for the workers [1,5].

**Figure 1:**
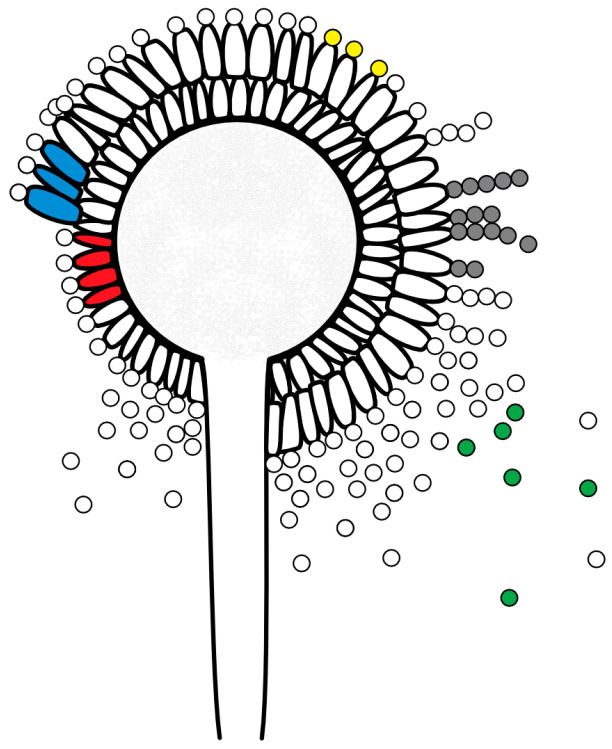
Schematic figure of an *A. flavus* conidiophore (fruiting body) with phialides (single-layer (uniseriate): red; double-layer (biseriate): blue), conidia (yellow), conidia chains (grey) and released conidia (green); © Tammo von Knoblauch.

The genus *Aspergillus* sp. belongs to the filamentous fungi [20]. It contains over 250 *Aspergillus* species currently divided into eight subgenera and twenty-two sections [21]. *Aspergillus* species are ubiquitous in the environment and are found worldwide [3,12,21,22,23]. They spread asexually via hydrophobic, resistant, airborne spores known as conidia, produced by fruit bodies known as conidiophores (Figure 1) [1,3,12,22,23]. *Aspergillus* sp. is not a bee-specific pathogen and can infect mammals, insects, birds, and plants [6,7,12,22,23,24,25].

Honey bees are associated with 25 *Aspergillus* species, independent of the disease stonebrood, although some of their descriptions are old and, therefore, possibly outdated [26]. Various species from the Fumigati, Flavi, and Nigri sections are responsible for the clinical picture of stonebrood [1,2,3,4,11,27]. *Aspergillus flavus* (section Flavi) is most frequently described in connection with stonebrood disease and has the highest virulence [1,2,3,4,11]. The two species *Aspergillus fumigatus* (section Fumigati) and *Aspergillus niger* (section Nigri), whose involvement in stonebrood infections is not clear, are isolated much less frequently [1,2,3,4,11,27]. Foley et al. were also able to isolate *A. phoenicis* (section Nigri) and *A. nomius* (section Flavi) and described both species as pathogenic for *A. mellifera* [3]. As molecular genetic methods for species differentiation were not available in earlier studies, it is not always clear whether the species mentioned in the literature are the species described. In 2007, for example, the species *A. niger* was reclassified so that *A. brasiliensis* is now listed as an independent but morphologically identical species based on molecular genetic analyses [28].

Depending on which species are involved, the stonebrood mummies change color when conidiophores (fruiting bodies) are formed (Figure 1) to green-yellowish if *A. flavus* is the predominant pathogen, grey-greenish if *A. fumigatus* is the predominant pathogen, and brown-black if *A. niger* is the predominant pathogen [1,5,12]. *A. flavus*, *A. fumigatus*, and *A. niger* can produce different mycotoxins [29]. While most mycotoxins either do not cause any damage to larvae or their effect is unknown, the aflatoxin B1 produced by *A. flavus* harms not only *A. mellifera* but also humans [5,26,30]. All three pathogens associated with stonebrood are zoonotic and can cause aspergillosis in humans [6,7,8]. In addition, the pathogen *Aspergillus* sp. can trigger an Extrinsic Allergic Alveolitis (EAA), also known as hypersensitivity pneumonitis, through the inhalation of antigens [9,10]. Therefore, stonebrood disease represents a health risk to beekeepers, and they must be able to recognize the disease in order to take appropriate precautionary measures.

*A. flavus* is generally a well-described fungus. The pathogen can not only infect Western honey bees (*A. mellifera*), but *A. flavus* infections have also been detected in the larvae of *Bombyx mori* (silkworm) [31,32] and of the wild bee species *Tetralonia lanuginosa* [33]. In adult insects, *A. flavus* infections have been detected in *Schistocerka gregaria* (Desert locust) [34], in *Blattella germanica* (German cockroach) [35], and in *Drosophila melanogaster* [36], among others.

There are large gaps in our knowledge of the pathogenesis of stonebrood in *A. mellifera* larvae. Our pathohistological study, conducted under controlled and standardized conditions, aims at closing these gaps and providing a better understanding of the pathogenesis and host–pathogen interaction of *A. flavus* infections. The results will be compared with existing studies, with a similar study about the chalkbrood pathogen *Ascosphaera apis*, and with studies about *Aspergillus flavus* infections in other insect larvae [31,32,33,37].

## 2. Materials and Methods

### 2.1. Bees and Aspergillus flavus Spores

Larvae from three healthy colonies of the western honey bee *A. mellifera* from the University of Copenhagen (Denmark) were used for the experiments. Colonies were headed by naturally mated, non-sister queens. The apiary (including the donor colonies) showed no clinical signs of fungal (including stonebrood), bacterial, or viral diseases in the year of the experiment and the following year. The good health status of the donor colonies was confirmed in the experimental infection assays by a mortality rate of 0% in the noninfected control group and the absence of *Aspergillus* sp. or otherwise infected larvae in the histological examinations of the control larvae.

The *Aspergillus flavus* strain (KVL14-113) was isolated from old pollen frames on a Sabouraud Dextrose Agar (SDA), subsequently cultured at 25 °C, and stored in the KVL culture collection of entomopathogenic fungi at the University of Copenhagen, Department of Plant and Environmental Sciences, Section of Organismal Biology. The morphology of the yellow-green conidia, the conidiophores, and the ITS sequence confirmed the species of *A. flavus*. Conidia were developed at an advanced growth stage and were accompanied by a color change in the colonies. The conidia were harvested from the SDA plate using a sterile spatula and transferred to a glass vessel containing 20 µL of sterile, demineralized water. A hemocytometer (Neubauer-improved, Marienfeld GmbH, Lauda-Königshofen, Germany) was used to quantitatively determine the conidia concentration. A viability and germination test was performed using previously published standard methods [5,38]. Briefly, 10 µL of a spore solution with 5 × 10^5^ spores per ml was plated on a Sabouraud Dextrose Agar (SDA) plate and incubated at 25 °C for 24 h. Thereafter, three cover slips were placed on the plate, and the percentage of germinated spores under each of the three cover slips was calculated under the microscope at 400× magnification.

### 2.2. Experimental Infection of Apis mellifera larvae

**Figure 2:**
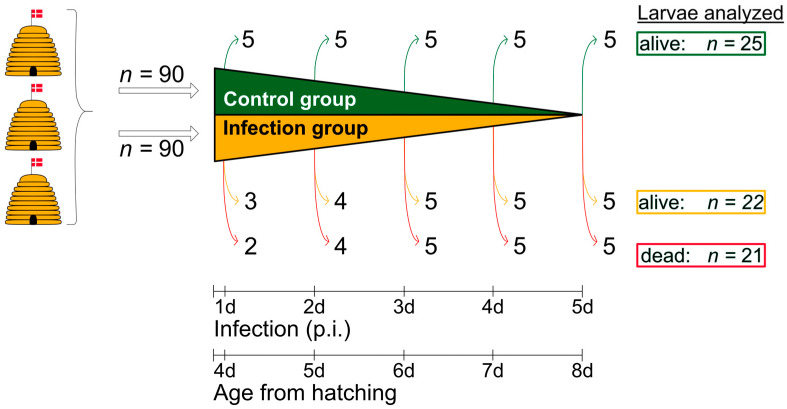
Schematic overview of the experimental infection assay. Larvae (*n* = 180) were taken from three donor colonies headed by naturally mated, non-sister queens and kept in an apiary near the University of Copenhagen (Denmark). Two groups were formed: one group consisting of uninfected control larvae (*n* = 90) and one group of infected larvae (*n* = 90). For macroscopic and histological examination, 25 larvae (5 × 5) were collected and analyzed from the control group, and 43 larvae (22 live larvae and 21 dead larvae) were analyzed from the infection group. The collection of live larvae (control group and infection group) was randomized; © Tammo von Knoblauch.

From each of the three *A. mellifera* bee colonies, 20 first instar larvae, approximately 12 h old, were collected in three rounds, giving a total of 180 larvae (*n* = 3 × 20 × 3). The age of the larvae was estimated based on their size. The larvae were randomly assigned to the two groups (control group and infection group) to minimize genetic influences on pathogenesis, which are already described for other diseases such as chalkbrood [38]. The larvae were transferred to 48-well plates 12 h after egg hatching. Each larva was placed in a separate well containing 10 µL Basic Larvae Food (BLF; 50% (*v*/*v*) royal jelly and 50% (*w*/*v*) aqueous solution in demineralized water (12% (*w*/*v*) D-fructose, 12% (*w*/*v*) D-glucose and 2% (*w*/*v*) yeast extract) and artificially reared in an incubator (Memmert, Schwabach, Germany) at 34 °C according to published standard methods [39]. The larvae were transferred daily to new wells with fresh food. The amount of food was increased by 10 µL per day. A randomly selected group (*n* = 90) was infected with *A. flavus* conidia three days after collection (i.e., as third instar larvae on day 4 after egg hatching) according to previously published standard methods [5]. For this purpose, 5 µL BLF with 1 × 10^5^ *A. flavus* conidia/mL BLF, which corresponds to an approximate conidia exposure of 5 × 10^2^ conidia per larva, was fed to each larva of the infection group. Five hours later, 15 µL conidia-free BLF was administered to achieve a total feed volume of 20 µL. The control group (*n* = 90) was fed 20 µL ((5 + 15) µL) conidia-free BLF. Conidia were fed once to the infection group, and on all subsequent days, both groups were fed only conidia-free BLF. The larvae/pupae were reared until day 14 p.i. to ensure that no late effects of the disease were overlooked. For this purpose, the larvae were placed in filter paper-lined wells after the first defecation, which is a marker for the fusion of the midgut and hindgut. In the colony, feeding would stop now and the cell would be capped. For further macroscopic and histological examinations, five larvae were collected daily from the control group between day 1 p.i. (third to fourth instar larvae) and day 5 p.i. (fifth instar larvae, also called prepupae). During the same period, five live and five dead larvae were collected daily from the infection group. The larvae were classified as dead when the movement of the spiracles stopped, indicating that the larvae stopped breathing. By then, they had also often lost their body elasticity or had become discolored, or fungal bodies and conidia were already protruding from the larval cuticle. Due to histological–technical problems, three infected live larvae and four infected dead larvae could not be further analyzed (Figure 2, day 1 p.i. and day 2 p.i.). In total, there were 25 living larvae from the control group, 22 living larvae from the infection group, and 21 dead larvae from the infection group (Figure 2). Larvae collected alive were sacrificed by CO_2_ exposure. All larvae were measured, documented photographically, and then transferred to 4% formaldehyde in PBS, pH 7.4, for overnight fixation at room temperature.

For a better understanding, the time of collection between day 1 and day 5 p.i. is given when describing the infected larvae and the control larvae. For the corresponding larval age (L; age from hatching), +3 days was added, as the time of infection was approximately 72 h after egg hatching. The age of the dead larvae could not be determined precisely, as they may have died shortly after the previous collection time. The time of death can, therefore, be up to 24 h earlier than the time of collection.

### 2.3. Preparation, Histological Measurements, and Evaluation

The macroscopic and histological preparations were carried out as stated in the recently published study on chalkbrood and can be read in detail there [37]. In summary, larvae fixed in 4% formaldehyde were embedded in paraffin according to standard protocols [40]. From the longitudinal median of the bee larvae, 2–3 µm thick serial sections were mounted on microscope slides, dried for 36 h at 30 °C, and then stained with hematoxylin–eosin and Grocott silvering [40].

**Figure 3:**
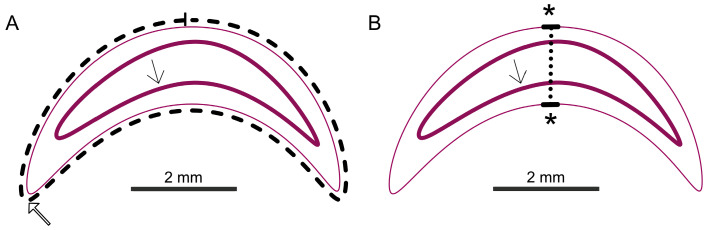
Schematic overview of the measurements (cuticle: thin pink line; cranial mouth opening: thick open arrow in (**A**); intestinal epithelium: thick pink line with thin arrow). The area measurement is shown in (**A**) (dashed line). The thickness measurement between the vertices (*) is shown in (**B**) (dotted line); © Tammo von Knoblauch.

Two median serial sections per larva were measured independently using Aperio ImageScope v12.3.3 software for the histological measurements. For the area measurement (in mm^2^), a measurement line was drawn along the entire cuticle (Figure 3A). The software automatically calculates the area based on the circumference. The thickness of the larvae (in mm) was measured by a line drawn between the vertices of the dorsal and ventral cuticle (thickest part of the larvae) (Figure 3B).

In the histological evaluation, germinating conidia and fungal mycelium were taken as evidence of a successful *A. flavus* infection. Only these larvae were assessed as reliably infected and are referred to as ‘infected’ in the following. As the infection status of all not reliably infected larvae is unclear, these larvae were excluded from further statistical analyses.

### 2.4. Statistical Analyses

Descriptive data analysis via a graphical analysis was performed using SPSS (Statistical Package for Social Science, Version 29.0.0.0, IBM, New York, NY, USA) to visualize the medians and interquartile ranges. The measurements were carried out twice per larva if possible. However, in a few cases, double measurement was impossible, because the sections were too heavily destroyed by the histological preparation. The samples were independent and not normally distributed. Therefore, statistical tests (two-sided Mann–Whitney U test) were performed within the control group and the reliably infected larvae (with histological signs) from the infection group at each time point. Statistical tests (Kruskal–Wallis tests and multiple pairwise comparisons according to Dunn’s procedure (two-sided test)) were also performed between the two groups. The significance level corrected by Bonferroni was 0.050.

## 3. Results

This study comprised a total of 180 larvae randomly assigned to two experimental groups (control group and infection group). Of those, a total of 68 larvae were collected and examined macroscopically and histologically (Figure 2). In total, 25 live larvae were collected from the control group, all of which did not show any signs of infection, neither macroscopically nor histologically. No dead larvae were collected from the control group. From the infection group, 43 larvae (alive: *n* = 22; dead: *n* = 21) were collected (Figure 2). Histologically, 19 of these collected larvae (live: *n* = 5; dead: *n* = 14) showed signs of infection with *A. flavus* in the form of germinating conidia and/or fungal mycelium. In the following, these larvae are described as infected. The remaining 24 larvae from the infected group (alive: *n* = 17; dead: *n* = 7) were macroscopically and histologically unobtrusive and indistinguishable from the larvae from the control group (see Appendix A for measurement data); therefore, we could not determine the cause of death of the seven dead larvae in the infection group. These larvae were not included in the statistical analyses due to their uncertain infection status (there may have been signs of infection in deeper sectional levels, or the signs of infection would have appeared later in sampling). They are referred to below as “not reliably infected”.

### 3.1. Macroscopic Findings in the Control Group and the Infection Group

**Figure 4:**
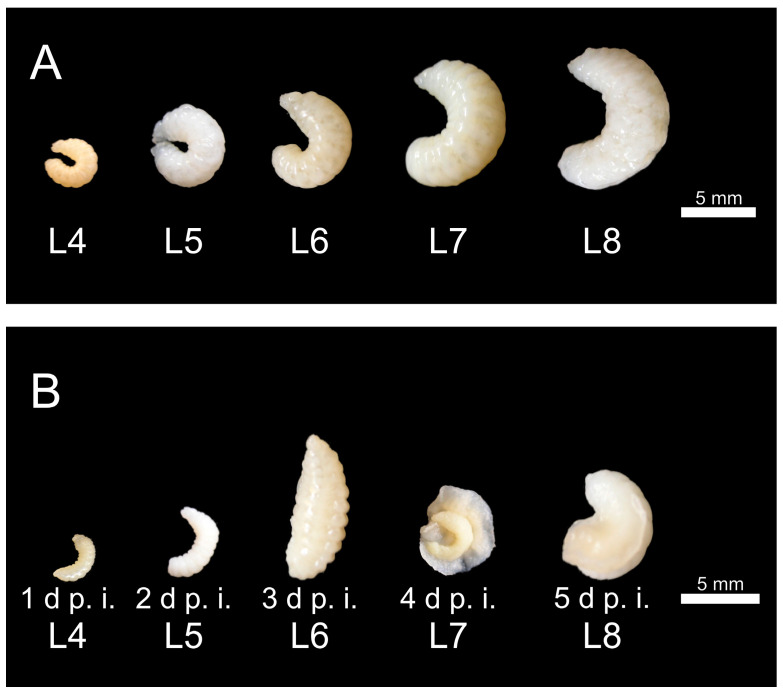
(**A**) Representative control larvae with an indication of larval age (Lx). The larvae were highly segmented and white-ivory colored with opaque to slightly translucent cuticles. They showed a loss of curvature with increasing age, reaching the largest expansion at 8 days of age (day 5 p.i.). (**B**) Larvae from the infection group that were found dead at the specified times (day p.i.) and collected for further analysis. The morphology and phenotype of the larvae that died at different times were highly variable, although they tended to be smaller and shriveled (1 d, 2 d, 4 d, 5 d p.i.) compared to the control larvae of the same age. On 3 d p.i., the dead larvae showed the widest size range, and even larvae much bigger than normal were observed (3 d p.i.). In some cases, the mycelium had broken through the cuticle and formed a spongy envelope around the larvae (4 d p.i., 5 d p.i.); © Tammo von Knoblauch.

**Figure 5:**
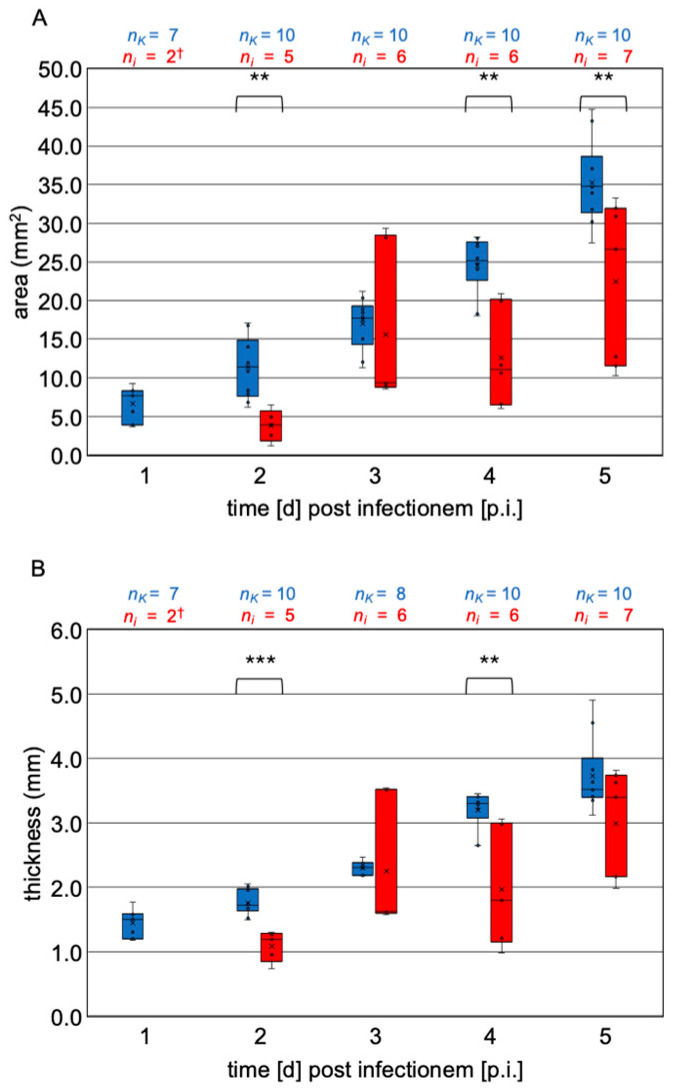
Boxplots (median, interquartile range, minimum, maximum, outliers) for the area in mm^2^ (**A**) and the thickness of the larvae in mm (**B**) at the collection times (1 d p.i. to 5 d p.i.). The control group (blue) and the infected larvae (red) are shown. Statistical analysis was performed using the Mann–Whitney U test. Statistical significances are represented by asterisks (** 0.001 < *p* ≤ 0.01; *** *p* < 0.001). The number of measured sections from the control larvae (*n_K_*) and the infected larvae (*n_i_*) is given. † Data for the infected larva collected alive from the infection group on day 1 p.i. are not shown because the number of analyzed sections (*n* = 2) did not allow for calculating the values for a boxplot and analyzing the data statistically (measurement data: see Table A1). © Tammo von Knoblauch.

The uninfected control larvae (*n* = 5 × 5), all collected alive at four to eight days of age, showed a substantial increase in size in the macroscopic examination up to day 5 p.i. (eight days of age) (Figure 4A). The histological measurements (area and thickness) and statistical tests (Kruskal–Wallis test and Dunn–Bonferroni tests) confirmed the increase in the size of the control larvae (Figure 5, Appendix A). While the larvae were strongly curved initially, the curvature decreased with age (Figure 4A). The color of the cuticle varied from opaque, dull, and ivory to translucent, shiny, and whitish (Figure 4A). The increase in size and macroscopic changes in the control larvae correspond to recently described findings for noninfected larvae from an independent study on the pathogenesis of chalkbrood [37].

Infected larvae (*n* = 19) were present daily from day 1 p.i. Macroscopically, the infected larvae had a very variable appearance with a tendency to be significantly smaller and to look shriveled compared to the control larvae (4 d, 5 d, 7 d p.i.) or enlarged and bloated (6 d, 8 d p.i.) (Figure 4B). They were slightly curved or elongated, and the tendency to elongate with increasing age (observed in the control larvae) was missing. The segmentation of the larvae was mostly indistinct. In some cases, the mycelium penetrated the cuticle and formed a sponge-like envelope around the larvae (Figure 4B, infection group 4 d p.i. and 5 d p.i.). The histological measurements (area and thickness) confirmed the macroscopic observation (Figure 5, Appendix A). The statistical analysis (Kruskal–Wallis test and Dunn–Bonferroni tests) of the infected larvae showed significantly larger larvae only on day 5 p.i. compared to day 2 p.i. (area: *p* < 0.01; thickness: *p* < 0.01; Table A2). The statistical comparison of the infected larvae with the larvae of the same age from the control group showed that the infected larvae were significantly smaller on day 2 p.i. (area: *p* < 0.01, thickness: *p* < 0.001), day 4 p.i. (area: *p* < 0.01, thickness: *p* < 0.01), and day 5 p.i. (area: *p* < 0.01).

### 3.2. Histological Findings in the Control Group and the Infection Group

**Figure 6:**
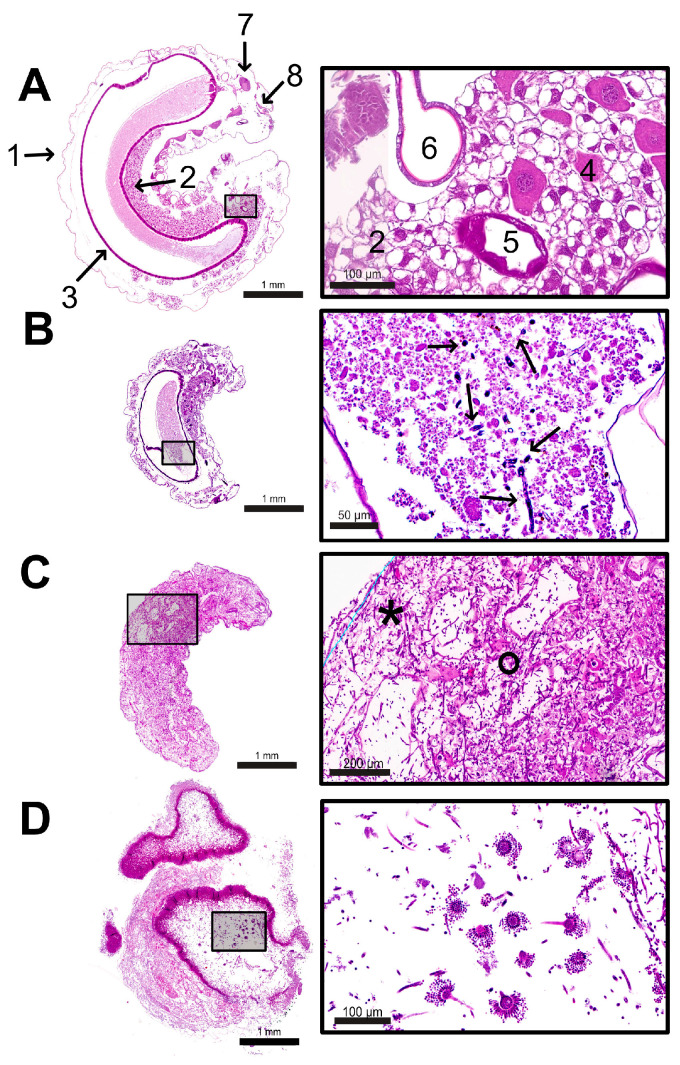
Overview images of larvae with magnifying zooms. (**A**) Overview and zoom of a median section of a control larva at the age of 2 days p.i. The larva is surrounded by the cuticle (1). The larval body is filled with fat body cells (2). Between the fat body cells, the midgut (3) lies centrally, consisting of a single row of intestinal epithelium. Ingesta lies within the midgut. Oenocytes (4) lie isolated between the fat body cells. Silk glands are ventral to the intestine (5), and parts of the tracheal system are frequently visible (6). The brain (7) and the mouth (8) are located cranially. (**B**) Overview and magnification (HE stain) of an infected larva at 1 d p.i., which was collected alive with signs of *A. flavus* infection. Germinating spores (arrows) in different stages were found in the midgut, inside the ingesta. (**C**) Overview and magnification (HE stain) of an infected larva at 2 d p.i. collected dead with signs of *A. flavus* infection. The tissue is interspersed and destroyed by hyphae (°), and some areas show autolysis (*). (**D**) Overview and magnification (HE stain) of an infected larva at 4 d p.i. The overview image of the larva shows mycelium concentrations, mainly in the area of the destroyed cuticle. Conidiophores (fruiting bodies) of *A. flavus* are visible; © Tammo von Knoblauch.

Our histological results from the control larvae (*n* = 25) show the cuticle as the outermost layer of the larval body. Inside lays the fat body (consisting of fat cells) with oenocytes located between the fat cells, the gut (consisting of foregut, midgut, and hindgut), and ven-trally of the gut silk glands and malpighian tubules (Figure 6A). Depending on the sectional level, the brain and other nerve cell assemblies (ganglia), the gonads, the musculature, and the tracheal system are visible (Figure 6A). While the foregut opened into the midgut through valves (*valvulae cardiacae*), the hindgut was not connected to the midgut since the histological examination period ended before the onset of metamorphosis, i.e., the time point when the midgut and hindgut fuse and the larvae defecate to empty the gut and prepare for metamorphosis. Due to the resulting impossibility of intestinal emptying, the midgut (depending on the section level) was mainly filled with ingesta, surrounded by the peritrophic matrix lining the midgut epithelium. These histological results correspond to recently described findings for noninfected larvae from an independent study on the pathogenesis of chalkbrood [37].

The infected larvae among the larvae collected alive or dead from the infection group showed different stages of infection (Table 1) indicated by the presence of germinating conidia, fungal mycelium, cuticle penetration, and even conidiophores. The observed conidia (purple in HE staining; black in Grocott silvering) had an average diameter of 3.5 µm (2–5 µm). They swelled to a diameter of 6 µm at the beginning of germination. The germinating hyphae (purple in the HE staining; black in the Grocott silver staining) were septate, still unbranched, and had an average diameter of 3.3 µm. The mature hyphae had a width between 2.5 µm and 4.8 µm and were septate and branched. The conidiophores had a diameter of 30 to 55 µm and were present with one or two rows of phialides. Ejected conidia had an average diameter of 3.5 µm (2–5 µm) (Figure 6D).

Of the twenty-two examined larvae that were collected alive from the infection group, five showed histological signs of infection: one each on days 1, 3, and 4 p.i. and two on day 5 p.i. (Table 1). A characteristic of these larvae was that the fungal mycelium was restricted to the midgut. The infected larvae showed germinating conidia only in a locally minimal area within the caudal half of the midgut (days 1, 4, and 5 p.i.; Figure 6B) or concentrated in a small area in the cranial midgut, behind the *valvulae cardiacae* (day 3 p.i.). The fungal mycelium was embedded in the ingesta (Figure 6B), had no direct contact with the epithelium, and did not penetrate the intestinal wall. There were no other visible deviations in the organs and tissues compared to larvae of the same age from the control group.

Of the twenty-one larvae examined that were removed dead from the infection group, fourteen were clearly infected according to the histological data: four on day 2 p.i., two on day 3 p.i., five on day 4 p.i., and three on day 5 p.i. (Table 1). Except for the two infected larvae from day 3 p.i., all other infected larvae were completely interspersed with fungal mycelium (Figure 6C) and revealed severe destruction of organs and tissues by *A. flavus*. Some larvae showed concentrations of the mycelium in the area of the former cuticle (Figure 6D) or formed ring-shaped formations within the whole larval body.

The two infected larvae from day 3 p.i. showed a different histological picture: Although the mycelium had also already broken through the intestinal epithelium, it had only spread in the caudal region of the larvae. The highest density of the mycelium was located adjacent to the intestinal epithelium of the caudal midgut in one larva and diffusely distributed in the caudal larval body in the other larva. There was no mycelium in the cranial third of the two larvae, and the tissue and organs appeared normal.

Penetration of the cuticle was not observed in any of the six examined larvae that were collected dead on days 2 and 3 p.i. However, in all five larvae collected dead on day 4 p.i. and in one of the three dead larvae collected on day 5 p.i., the cuticle was penetrated by fungal mycelium (Table 1).

The effects of autolytic processes and tissue destructions were visible in all bodies of the infected larvae collected dead, albeit to varying degrees (Table 1): In the bodies of the examined larvae collected dead on days 2 and 3 p.i., autolytic processes were only partially visible (Figure 6C), while the bodies of the larvae collected dead on days 4 and 5 p.i. were already (almost) completely destroyed. For instance, in three of the five dead larvae collected on day 4 p.i., the larval contour, tissue, and organ-specific structures could not be identified histologically.

Conidiophores could not be observed in any larva except for three of the five infected larvae collected dead on day 4 p.i. (Figure 6D). These fruiting bodies were mainly present ventrally outside the former larval body.

The larvae/pupae observed beyond the onset of metamorphosis until day 14 p.i. showed no signs of stonebrood or any further undescribed effects, suggesting that defecation cleansed the larvae of the remaining non-germinated spores or that the germination of spores was no longer possible in the pupal phase.

In summary, 44% (*n* = 19) of the larvae collected from the infection group (*n* = 43) showed histological signs of infection from day 1 p.i. onwards (referred to as infected). Of these larvae, 74% (*n* = 14) were collected dead. The pathological findings showed an extremely aggressive course of *A. flavus* growth (Table 1): In the infected larvae collected alive, the only signs of infection were germinating conidia in small areas of the midgut. In the infected larvae that were collected dead, the intestinal epithelium and at least two-thirds of the larvae were already penetrated by mycelium. In contrast, all the larvae from the control group were collected alive and showed no signs of infection or other histological abnormalities.

## 4. Discussion

*Aspergillus* is a large genus of fungi that is of great importance in medicine due to the ubiquitous occurrence of (human) pathogenic species worldwide [6,7,22,23,24,25]. *A. flavus*, more rarely also *A. fumigatus* or *A. niger*, can infect both larvae and adults of the Western honey bee *A. mellifera* and cause the clinical picture of stonebrood [1,2,3,5]. In this experimental study, larvae of *A. mellifera* were orally infected with *A. flavus* to obtain infected larvae of different ages (four- to eight-day-old bee larvae) that could be examined histopathologically to unravel the pathogenesis of larval stonebrood infection.

### 4.1. Infection Rate

All the larvae collected for macroscopic and histological examination (*n* = 43) from the infection group were infected with a dose of 5 × 10^2^ *A. flavus* conidia three days after egg hatching. Of these, however, only 19 larvae showed histological signs of infection. One possible explanation is that the conidia dose was simply insufficient to infect all larvae since the LC_100_ was not determined before conducting this experiment. It is also possible that conidia or hyphae in deep section levels may have been overlooked since only median section levels were analyzed histologically. However, several paramedian serial sections were obtained per larva, which were also analyzed for hyphae or germinating spores in the infection group and which did not show any signs of infection. It is possible that an infection could have developed at a later larval stage in the larvae collected alive that did not show any fungal growth. The individual immunity, genetic resistance, or effects of the microbiome may play a role in the fact that the infection did not break out [41,42,43]. Even though there is no robust statement about the infection rate due to the study design, it can be assumed that the infection rate is at least 44% (*n* = 19) or possibly even higher.

### 4.2. Larval Growth

As expected, the histological measurements of the control group show a steady increase in growth (area: *p* < 0.001; thickness: *p* < 0.001). Growth in the infected larvae is statistically detectable between day 2 p.i. and day 5 p.i. (area: *p* < 0.01; thickness: *p* < 0.01). However, the infected larvae are statistically significantly smaller than the larvae from the control group on day 2 p.i. (area: *p* < 0.01, thickness: *p* < 0.001), day 4 p.i. (both: *p* < 0.01), and day 5 p.i. (area: *p* < 0.01), suggesting that *A. flavus* infections may negatively impact larval growth. As food intake was not controlled in this experimental setup, the reduced growth may also be due to decreased food intake; however, no histological abnormalities indicating reduced feed intake were observed in the larvae from the infection group.

### 4.3. Pathogenesis of Stonebrood

**Figure 7:**
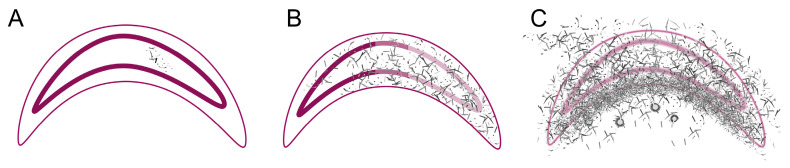
Schematic overview of the different stages of infection (cuticle: thin pink line; intestinal epithelium: thick pink line). (**A**) Phase 1—Germination and spreading of *A. flavus* hyphae (grey-black) in the midgut. (**B**) Phase 2—Spreading of *A. flavus* hyphae (grey-black) within the larval body of the dead larva. (**C**) Phase 3—Penetration of the cuticle with the formation of conidiophores (balls); © Tammo von Knoblauch.

As the first infected larvae could already be collected on day 1 p.i., it is safe to state that the incubation period of *A. flavus* is or can be less than 24 h. The first dead larvae with signs of infection already appeared on day 2 p.i. This is consistent with the experiments by Burnside, who described the death of the larvae from day 2 p.i. onwards and who wrote about the rapid spread of *A. flavus* [1]. Our study shows that the pathogenesis of *A. flavus* can be divided into three phases (Figure 7). In the first phase (establishment phase; Figure 7A), the fungus germinated within the intestine and was localized in a small area. All infected larvae collected alive were in this phase. In the second phase (spreading phase; Figure 7B), the mycelium penetrated the intestinal epithelium and spread throughout the larval body. The tissue and organs of the larvae were destroyed mainly by the mycelium and autolysis; in some cases, only fungal mycelium remained. The cuticle was not perforated. This phase was only observed in larvae that were collected as dead larvae. The last phase (distribution phase; Figure 7C) was characterized by the penetration of the cuticle with the subsequent formation of conidiophores. All larvae belonging to this phase were already dead at the time of collection. Burnside’s experiments and our study observed that the mycelium only breaks through the cuticle after the death of the larva, forming the typical stone brood mummies [1]. As in the macroscopic changes shown here, Burnside also observed changes in appearance and texture between death and mycelial expansion outside the larval body [1].

According to our observations, the larva dies between phase 1 and phase 2, possibly even before penetration of the intestinal epithelium, which could not be observed. This indicates that mechanical damage by the mycelium is unlikely to be the cause of death. Since *A. flavus* can produce mycotoxins such as aflatoxin B1 under favorable conditions, we assume that the larvae may have died due to the toxin exposure and that the fungus then spread saprophytically [1,26,30,44,45]. Two further studies have shown that aflatoxin can be produced in larvae and may damage bees, which could support our theory [30,44]. Toxin presence should be analyzed in further studies to confirm this theory.

In 33% (*n* = 7) of the dead larvae from the infection group, we did not observe any histological signs of infection. However, we cannot rule out that *A. flavus* may have developed at deeper sectional levels and produced toxins harming the larva without extensive fungal growth. This again points to the necessity to analyze *A. flavus* toxin production and activity in further studies.

The absence of transitional stages, the presence of phase 2 already at day 2 p.i., and cases in which the larva was completely interspersed with mycelium, while, at the same time, autolysis was not yet well advanced, indicated that the course of infection of *A. flavus* is very fulminant and aggressive, which was confirmed in the study by Vojvodic et al. [46]. *A. flavus* also appears to infect every tissue and every organ. However, it should be noted that the hyphae only spread outside the midgut after death. It was not always possible to distinguish between destruction by fungal mycelium and autolysis with subsequent mycelial growth. In his study, Burnside describes that soft tissue in adult bees’ abdomen, thorax, and head is immediately attacked after death [1]. He describes that malpighian tubules are usually not affected until the death of the bees and that tracheae are not affected even after death due to the dry conditions and the strong tracheal wall [1]. In our study, we did not observe any exceptions in larvae. This may also be because the organs of larvae are not yet as mechanically resistant as those of adult bees. Our study clearly showed that the cuticle is only penetrated at a late stage, to be more precise, after the entire larval body has been overgrown with fungal mycelium. This step then initiates phase 3, the distribution phase, with the formation of conidiophores. The localization of the conidiophores outside the larval body or, in a few cases and the case of severely damaged larvae, also inside, close to the former cuticle, suggests that *A. flavus* seeks exposed sites (exposed to the air) for optimal distribution of the conidia. Bailey described this already [12]. The shape of the conidiophores (single- or double-row with philadia up to the stalk) shows that the infection is indeed an *A. flavus* infection. The third phase is, therefore, also responsible for the clinical picture of stonebrood: mummified larvae in different colors, depending on the Aspergillus species. *Aspergillus flavus* appears to spread far outside the (former) larval body. This was also seen in the macroscopic images showing larvae with a thick sponge-like coating (Figure 4: day 4 p.i. and day 5 p.i.). The larger surface area and continued growth outside the larval body (without direct nutrient supply) could explain why mummified stonebrood larvae can infiltrate the brood cells and, as often described, are difficult to remove from the cells [5]. As already observed in Burnside’s study, a pseudocuticle forms in the area of the former cuticle [1]. The ring-shaped mycelial concentrations within the larval body are possibly responsible for the typically hard stonebrood mummies. Burnside described that the ventriculus of infested adult bees requires considerably more pressure when pressed under a cover glass and that mycelial concentrations in digestive organs and tissues are responsible for increased firmness [1]. We assume he described the ring-shaped mycelial concentrations within the larval body here. The third phase also represents a health risk for the beekeeper, as the spreading spores can cause aspergillosis or hypersensitivity pneumonitis, especially in the respiratory tract [6,7,8,10]. Therefore, it is advisable to wear respiratory protection in clinically affected colonies.

### 4.4. Comparison of Stonebrood and Chalkbrood

A comparison with a second fungal brood disease, chalkbrood, reveals fundamental differences in the pathogenesis of the two fungal infections (Table 2): The incubation period of *Aspergillus flavus* with less than 24 h is considerably shorter than that of *Ascosphaera apis* (48–72 h) [37]. While *Ascosphaera apis*-infected larvae can still live when fungal mycelium spreads outside the midgut, this is impossible with *Aspergillus flavus*-infected larvae. The death of the larvae, which is induced by damage to the tissue and organs in an *Ascosphaera apis* infection, may be caused by toxin activity in an *A. flavus* infection. Both *Ascosphaera apis* and *Aspergillus flavus* form a pseudocuticle in the area of the former cuticle, and they appear to primarily form fruiting bodies outside the former larval body [37]. In contrast to *Aspergillus flavus*, *Ascosphaera apis* spreads much less outside the (former) larval body [37].

### 4.5. A. Flavus Infections in Other Insect Species

A comparative analysis of the literature shows that *A. flavus* infections can also occur in other insect species. In one study, larvae of the wild bee species *Tetralonia lanuginosa* showed mortality rates of 48%, which were associated with aflatoxin production [33]. The silkworm *Bombyx mori* can also be infected by *Aspergillus flavus* (and other *Aspergillus* species) [31,32]. In the study by Kumar et al., larvae of the silkworm *Bombyx mori* were topically infected with *A. flavus* on the cuticle [31]. Although this is a different type of infection, the germination on the cuticle was observed as early as 6 h, but larval death was only observed 4–5 days after infection [31]. The later death may be related to the fact that the cuticle is very resistant. There are no other comparable infection studies in other insects.

### 4.6. Outlook and Conclusions

The results presented here do not allow for any conclusions on the pathogenesis of *A. flavus*-infected adult honey bees or the role of other pathogens associated with stonebrood, such as *A. fumigatus* and *A. niger*. These must be investigated further to gain an overall understanding of the stonebrood disease. The early death of the larvae—when the mycelium has not yet spread outside the midgut—presumably leads to the fact that honey bee workers can still remove the larvae, and the disease, therefore, remains undetected by the beekeeper. *A. flavus* continues to spread out in a necrotrophic phase after the death of the larva and primarily forms conidiophores outside the (former) larval body, which are essential for horizontal spread. When a colony is too weak to remove the larvae at an early stage, it is probably much more difficult for the worker bees at this stage to remove the mycelium, as it infiltrates the wall of the brood cell [1,5]. In conclusion, this study showed that *A. flavus* can establish rapidly (within 24 h) in experimentally infected bee larvae and can cause death of the larvae within 48 h. Since the larvae died very soon after the onset of infection, we hypothesize that the death of the larvae may be caused by toxin exposure. At this time, the larvae can be removed by adult bees. After the death of the larva, *A. flavus* grows necrotrophically and eventually spreads outside the larvae to form conidiophores.

## Figures and Tables

**Table 1:** Overview of the most important histological characteristics of infected larvae collected alive or dead from the infection group at the individual time points.

	1 d p.i.	2 d p.i.	3 d p.i.	4 d p.i.	5 d p.i.
infected larvae/living larvae *	1/3	0/4	1/5	1/5	2/5
mycelium localization	midgut	-	midgut	midgut	midgut
	(*n* = 1/1)		(*n* = 1/1)	(*n* = 1/1)	(*n* = 2/2)
					
infected larvae/dead larvae **	0/2	4/4	2/5	5/5	3/5
mycelium localization	-	complete larval body	caudal part of larval body	complete larval body	complete larval body
		(*n* = 4/4)	(*n* = 2/2)	(*n* = 5/5)	(*n* = 3/3)
cuticle penetration	-	no	no	yes	yes
				(*n* = 5/5)	(*n* = 1/3)
autolysis	-	partially	partially	complete	complete
		(*n* = 2/4)	(*n* = 1/2)	(*n* = 5/5)	(*n* = 3/3)
conidiophores	-	no	no	yes	no
		(*n* = 0/4)	(*n* = 0/2)	(*n* = 3/5)	(*n* = 0/3)

Note: *, number of infected larvae among the larvae collected alive from the infection group; **, number of infected larvae collected dead from the infection group.

**Table 2:** Differences in the pathomechanisms of the two mycological brood diseases of *A. mellifera*.

	Stonebrood	Chalkbrood
Pathogen	*Aspergillus flavus*	*Ascosphaera apis*
Incubation period	<24 h	48–72 h
Localization germination	whole midgut	caudal part of midgut
Time of death	from 24 to 48 h p.i.	from 72 h

## Data Availability

The original contributions presented in this study are included in the article. Further inquiries can be directed to the corresponding authors.

## Appendix A

**Table A1:** Measurement data for area and thickness were divided into a control group and an infection group—with and without histological signs.

**Collection Time Point**	**Control Group**		**Infection Group**			
	**Noninfected**		**With Histological Signs of Stonebrood (Infected)**		**Without Histological Signs of Stonebrood**	
	**Area Median in mm^2^ (min–max)**	**Thickness Median in mm (min–max)**	**Area Median in mm^2^ (min–max)**	**Thickness Median in mm (min–max)**	**Area Median in mm^2^ (min–max)**	**Thickness Median in mm** **(min–max)**
1 d p.i.	7.7 (3.7–9.2)	1.5 (1.2–1.8)	- * (4.0–4.8)	- * (1.3–1.3)	6.0 (1.5–8.2)	1.2 (0.8–1.3)
2 d p.i.	11.4 (6.2–17.1)	1.7 (1.5–2.0)	3.9 (1.2–6.5)	1.2 (0.7–1.3)	11.0 (3.9–15.2)	2.1 (1.3–2.3)
3 d p.i.	17.7 (12.0–21.2)	2.3 (2.2–2.5)	9.3 (8.5–29.3)	1.6 (1.6–3.5)	20.4 (7.3–27.7)	2.7 (1.4–3.5)
4 d p.i.	25.1 (18.1–28.2)	3.3 (2.6–3.5)	11.1 (6.0–20.9)	1.8 (1.0–3.1)	20.1 (9.1–26.7)	2.7 (1.8–3.6)
5 d p.i.	34.7 (27.4–44.7)	3.5 (3.1–4.9)	26.7 (10.3–33.3)	3.4 (2.0–3.8)	32.4 (19.2–41.5)	3.9 (3.1–4.6)

Note: *, The median area and median thickness for the infected larva collected alive from the infection group on day 1 p.i. are not provided because the number of analyzed sections (*n* = 2) did not allow for their calculation.

**Table A2:** Statistical data from the Kruskal–Wallis test and subsequent post hoc tests (Dunn–Bonferroni tests) for significant size differences within the control and infection groups. Non-statistical significances are abbreviated with n.s.

**Collection Time Point**	**Control Group** **(Noninfected)**		**Infection Group** **(With Histological Signs of Chalkbrood;** **Infected)**	
	**Area**	**Thickness**	**Area**	**Thickness**
Kruskal–Wallis test	*p* < 0.001	*p* < 0.001	*p* < 0.01	*p* < 0.01
Dunn–Bonferroni tests				
day 1 vs. day 4	*p* = 0.001	*p* < 0.001	-.*	-*
day 1 vs. day 5	*p* < 0.001	*p* < 0.001	- *	- *
day 2 vs. day 4	*p* = 0.014	*p* = 0.004	n.s.	n.s.
day 2 vs. day 5	*p* < 0.001	*p* < 0.001	*p* < 0.01	*p* < 0.01
day 3 vs. day 5	*p* = 0.011	n.s.	n.s.	n.s.
day 4 vs. day 5	n.s.	n.s.	n.s.	n.s.

Note: *, Statistical data are not provided for the infected larva collected alive from the infection group on day 1 p.i. because the number of analyzed sections (*n* = 2) did not allow for statistical analyses.
